# Supplementary material for: Elucidating the impact of bacterial lipases, human serum albumin, and FASII inhibition on the utilization of exogenous fatty acids by Staphylococcus aureus
Source: mSphere. 2023 Nov 28;8(6):e00368-23. doi: 10.1128/msphere.00368-23 (PMC10732024; doi:10.1128/msphere.00368-23)
Supplement: Supplemental Figures — Figures S1 to S5. [file msphere.00368-23-s0001.pdf]

Supplemental Material for

Elucidating the Impact of Bacterial Lipases, Human Serum Albumin, and FASII Inhibition on the Utilization of Exogenous Fatty Acids by *Staphylococcus aureus*

Emily L. Pruitt,[a] Rutan Zhang,[b] Dylan H. Ross,[b,#] Nathaniel K. Ashford,[c,#] Xi Chen,[d,#] Francis Alonzo III,[d,#] Matthew F. Bush,[a] Brian J. Werth,[c] Libin Xu,[b,*]

[a] Department of Chemistry, University of Washington, Seattle, Washington, USA

[b] Department of Medicinal Chemistry, University of Washington, Seattle, Washington, USA

[c] Department of Pharmacy, University of Washington, Seattle, Washington, USA

[d] Department of Microbiology and Immunology, Loyola University Chicago- Stritch School of Medicine, Maywood, Illinois, USA

Running Title: Exogenous fatty acids utilization by *S. aureus*

*, Address correspondence to Libin Xu, libinxu@uw.edu.

Present addresses:

[#]Dylan H. Ross, Pacific Northwest National Laboratory, Richland, Washington, USA

[#]Nathaniel K. Ashford, University of Washington School of Medicine, Seattle, Washington, USA

[#]Francis Alonzo III, Department of Microbiology and Immunology, University of Illinois- College of Medicine at Chicago, Chicago, Illinois, USA

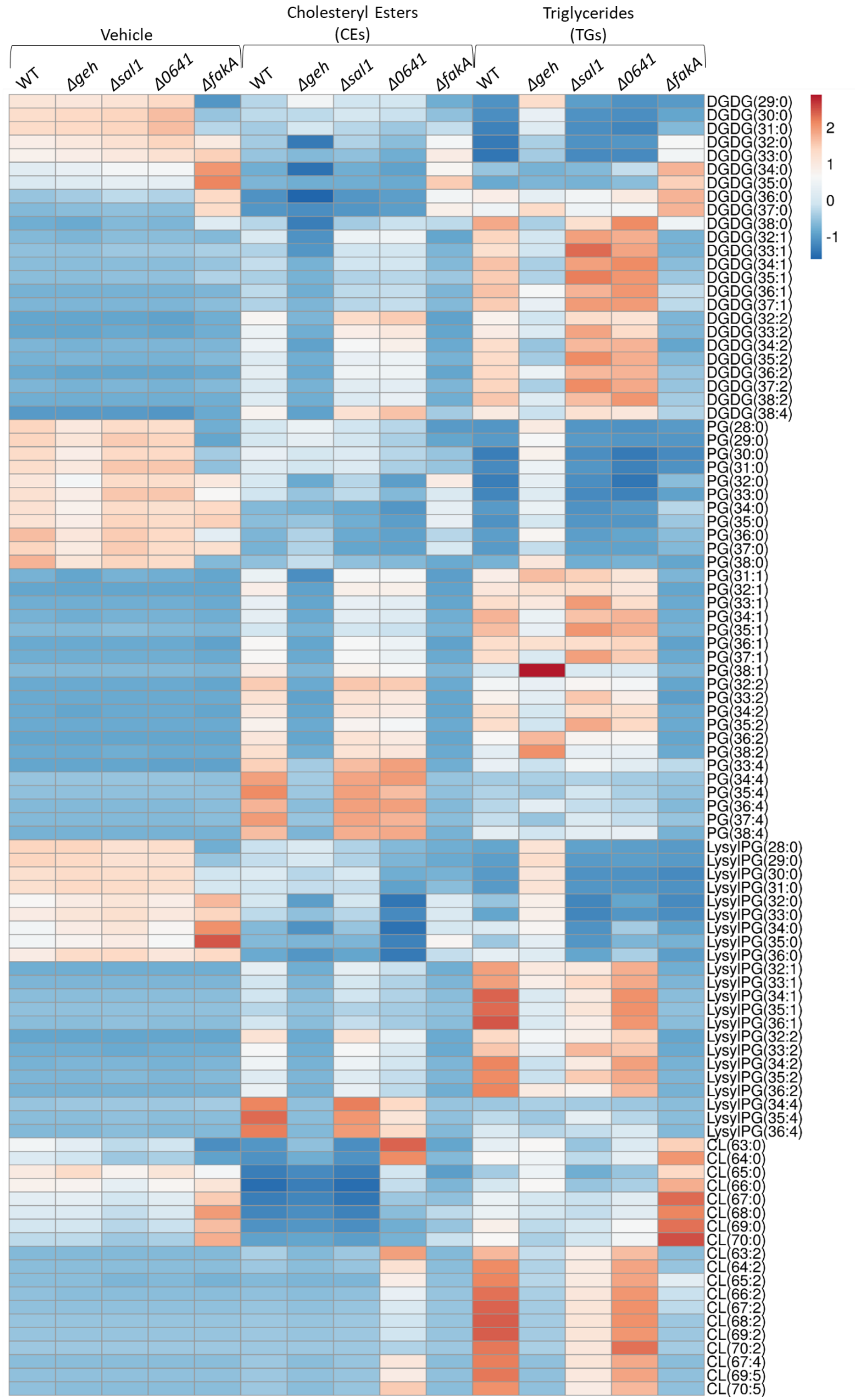

Figure S1. Relative abundances of lipids of WT (USA300 LAC) and *geh*-, *sal1*-, *0641*-, or *fakA*-knockout mutant strains grown in TSB or TSB + cholesteryl esters (CEs) or triglycerides (TGs) containing C18:1, C18:2, or C20:4 at 100 μM for each lipid. Results are row-centered and scaled by unit variance scaling. N = 4 per group.

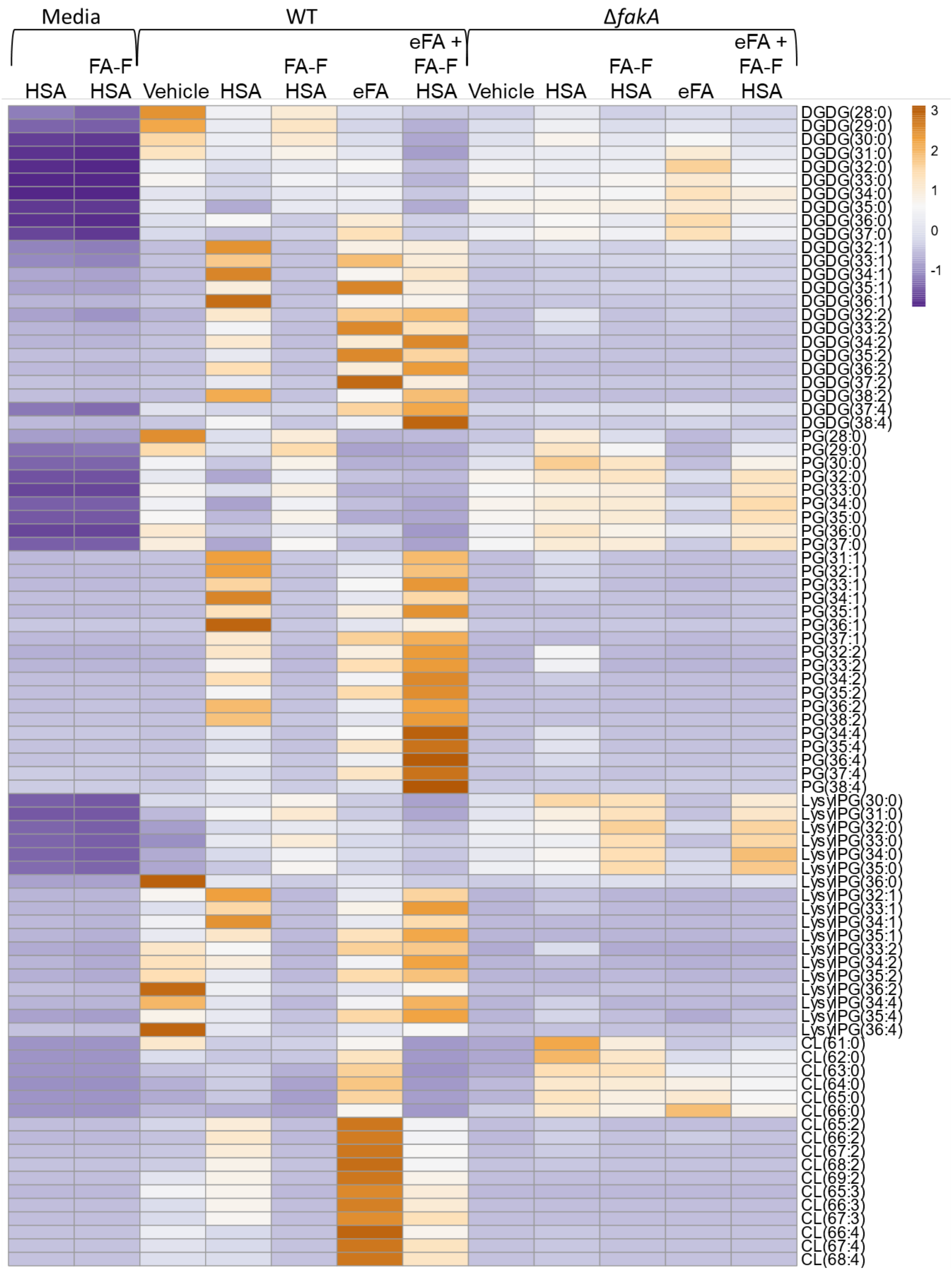

Figure S2. Effect of fatty acid-containing HSA and fatty acid-free (FA-F) HSA at 10 mg/mL on the incorporation of eFAs mixture (oleic acid 18:1, linoleic acid 18:2, and arachidonic acid 20:4) into bacterial lipids. Results are row-centered and scaled by unit variance scaling. N = 4 per group.

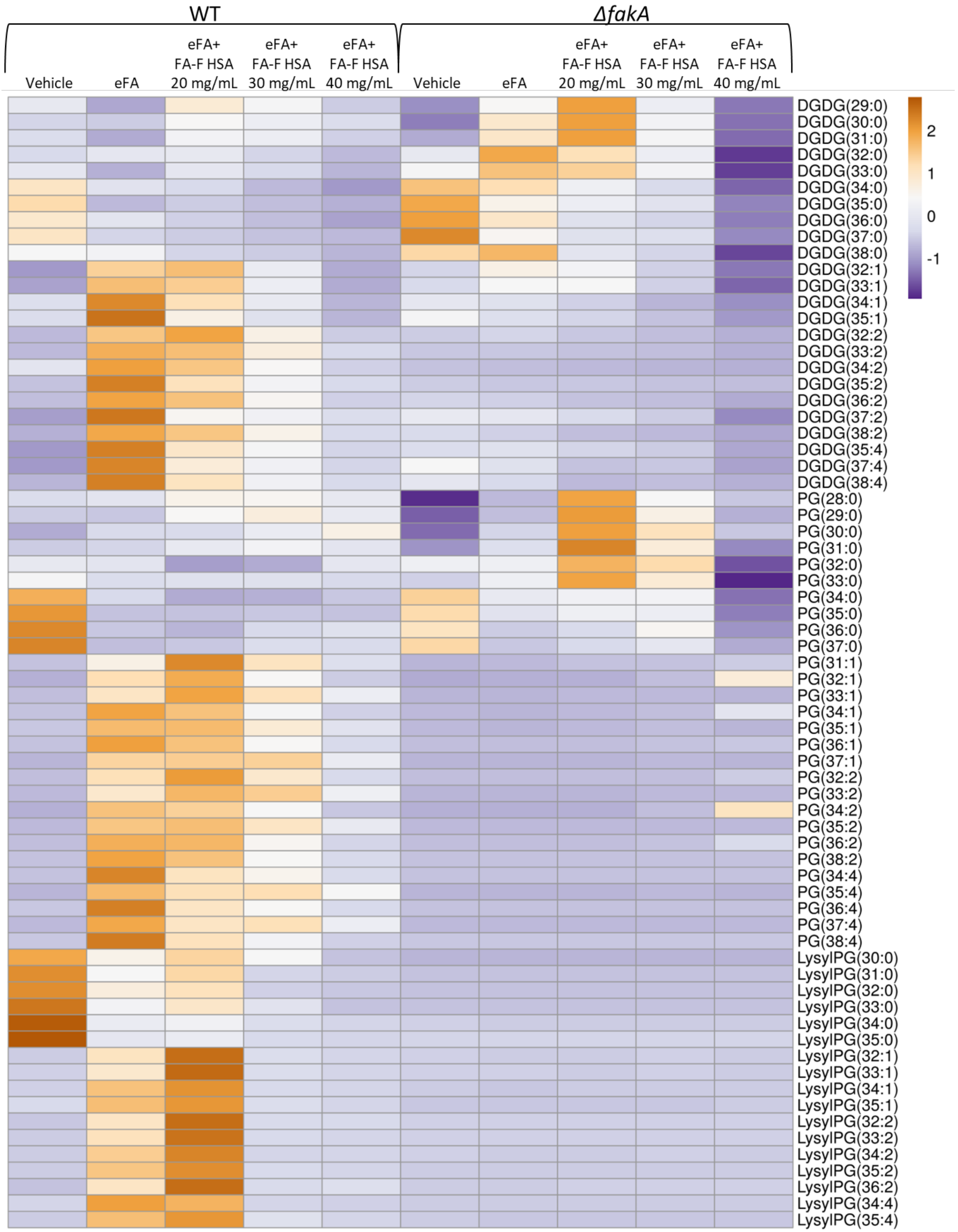

Figure S3. The effect of increasing concentrations of FA-F HSA on the incorporation of eFAs mixture (oleic acid 18:1, linoleic acid 18:2, and arachidonic acid 20:4) into bacterial lipids. Results are row-centered and scaled by unit variance scaling. N = 4 per group.

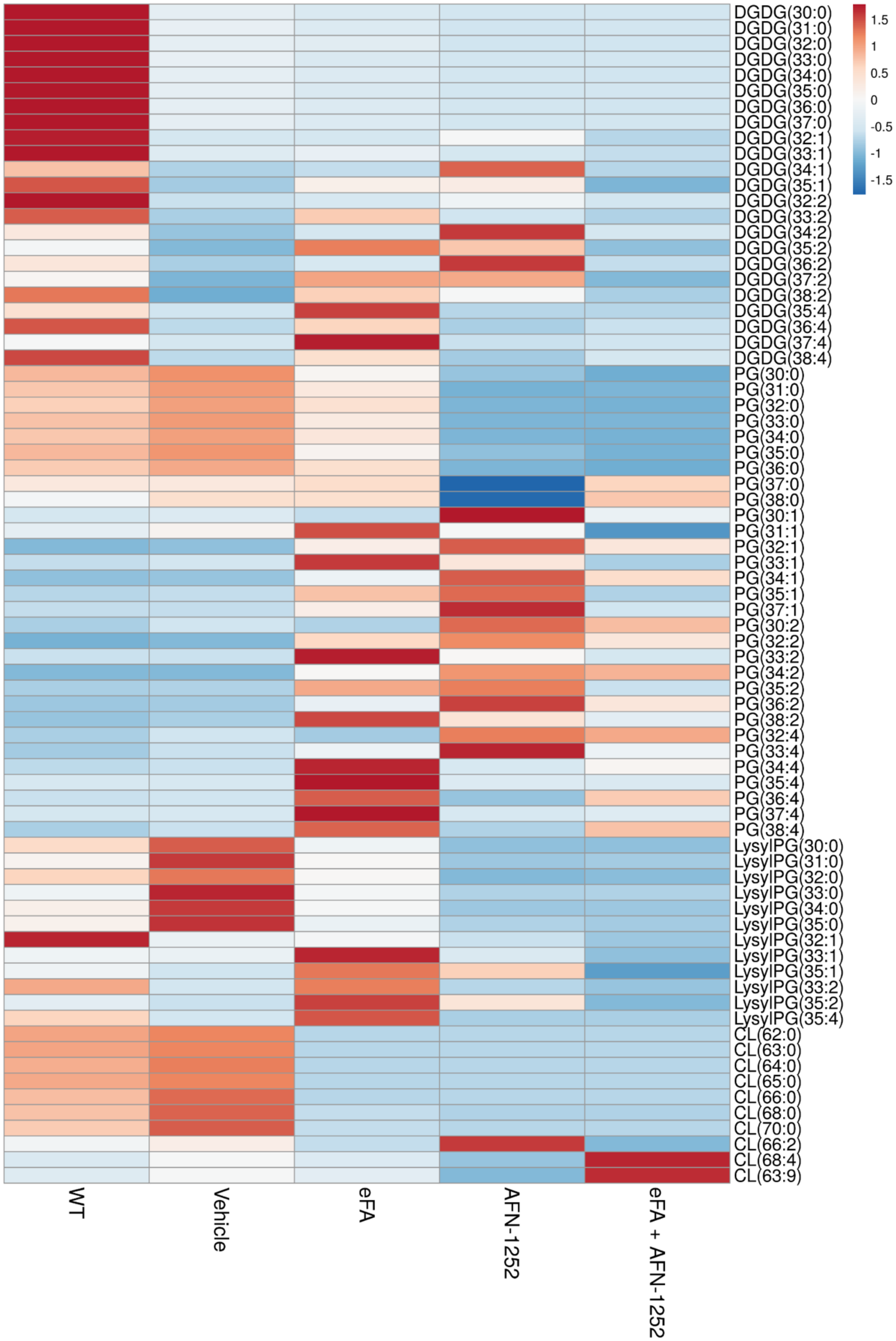

Figure S4. Effect of AFN-1252 on the incorporation of eFA standards containing fatty acids 18:1, 18:2, and 20:4 into various lipid classes in *S. aureus*. Results are row-centered and scaled by unit variance scaling. N = 3-4 per group.

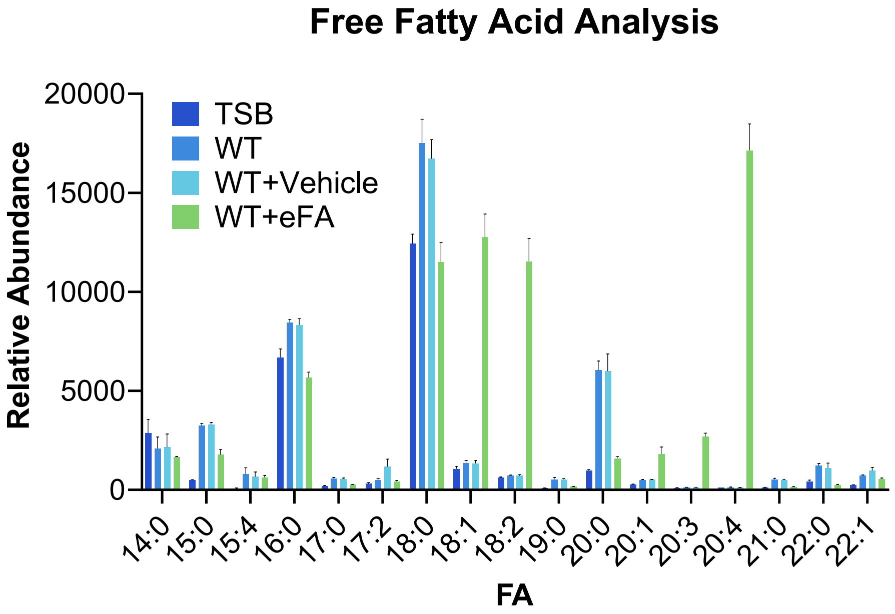

Figure S5. Relative abundances of fatty acids identified in the same volume of TSB and in WT strain grown in TSB with or without eFA standards containing fatty acids 18:1, 18:2, and 20:4. N=3-4 per group.
